# Nailfold capillaroscopic assessment in pediatric patients with autoimmune uveitis: a case–control study

**DOI:** 10.1007/s10067-024-07183-0

**Published:** 2024-10-27

**Authors:** Maha S. I. Abdelrahman, Dalia Tohamy, Naglaa S. Osman, Mohamed G. A. Saleh

**Affiliations:** 1 Department of Rheumatology, Rehabilitation, and Physical Medicine, Faculty of Medicine, Assiut University, Assiut, 71515 Egypt; 2 Department of Ophthalmology, Faculty of Medicine, Assiut University, Assiut, Egypt; 3 Pediatric Allergy, Immunology, and Rheumatology, Children Hospital, Assiut University, Assiut, Egypt

**Keywords:** Pediatric autoimmune uveitis, Nailfold capillaroscopy, Microcirculation

## Abstract

Uveitis is a major cause of visual impairment. Most uveitis cases have autoimmune etiology. Pediatric autoimmune uveitis may be associated with systemic diseases such as juvenile idiopathic arthritis or may arise as an isolated disorder. It may be accompanied by retinal vasculitis due to retinal microcirculation involvement. Nailfold capillaroscopy, a digital microscope, is a non-invasive tool for systemic microcirculation evaluation. We aimed to evaluate systemic microcirculation abnormalities in pediatric autoimmune uveitis. Twenty-five patients with pediatric autoimmune uveitis and 21 healthy children underwent detailed capillaroscopic evaluation. We assessed capillary density/mm, capillary morphology, capillary dimensions, and the presence or absence of microhemorrhages and avascular areas. The mean age of the study and control groups was 11.24 ± 3.03 and 9.9 ± 4.17 years, respectively. Most included patients had isolated uveitis and juvenile idiopathic arthritis (64% and 24%, respectively). The predominant uveitis subtype in the study was anterior uveitis (48%). A significant difference was found between cases and controls regarding mean capillary density (*p*-value = 0.0003) and the number of subjects having capillary density less than 7 (*p*-value = 0.002). Other capillaroscopic abnormalities did not show any significant difference between the studied groups. Mean capillary density did not correlate significantly with age, disease duration, or acute phase reactants. Children with autoimmune uveitis, whether isolated or as a part of systemic disease, may have systemic microcirculation involvement.

**Table:** 

**Key Points** • *Idiopathic autoimmune uveitis is not always an isolated intraocular condition.* • *Systemic microcirculation involvement may occur in pediatric autoimmune uveitis, even in cases with isolated uveitis.* • *Nailfold capillaroscopy showed that capillary density in children with autoimmune uveitis is significantly reduced compared to healthy controls.*

**Key Points**

• *Idiopathic autoimmune uveitis is not always an isolated intraocular condition.*

• *Systemic microcirculation involvement may occur in pediatric autoimmune uveitis, even in cases with isolated uveitis.*

• *Nailfold capillaroscopy showed that capillary density in children with autoimmune uveitis is significantly reduced compared to healthy controls.*

## Introduction

Uveitis is a vision-threatening disorder, either directly or via causing complications [1]. Around 2 million individuals worldwide are diagnosed with uveitis [2]. Uveitis is categorized etiologically as either infectious or noninfectious [3]. Autoimmunity is implicated in the pathogenesis of noninfectious uveitis [4]. In autoimmune uveitis (AU), tissue damage or microbial triggers enhance the production of antigen-specific T cells and autoantibodies with a pathogenic function [5].

AU is implicated in most uveitis cases. In Western countries, AU accounts for about 10 to 15% of visual loss cases [6]. It affects all age clusters, so it can cause a significant socioeconomic problem [7]. AU, in particular, may be a warning sign of underlying systemic disease and may lead to significant visual impairment up to visual loss when the posterior segment of the eye is involved [8].

Pediatric AU may be associated with systemic diseases such as juvenile idiopathic arthritis (JIA). In most cases, no cause could be detected; these cases are considered isolated idiopathic uveitis [9, 10]. About 80% of pediatric idiopathic uveitis cases have a coexisting retinal microcirculation involvement, manifested as retinal vasculitis, with subsequent worse visual prognosis [11].

Nailfold capillaroscopy (NFC) is a non-invasive tool that can be applied in children and adults and provides a unique microcirculation assessment. Abnormal capillaroscopy is helpful in diagnosis, monitoring disease activity, and evaluating response to therapy, particularly with high-magnification video systems that allow accurate visualization of capillary abnormalities [12]. NFC has a value in the monitoring of disease activity in some autoimmune connective tissue diseases such as systemic sclerosis or the cutaneous domain of dermatomyositis, whereas in the other CTDs and even less in other rheumatic diseases, the value of the monitoring is less established [13].

We aimed to evaluate NFC results in pediatric AU cases compared to healthy children.

## Patients and methods

### Study subjects

Initially, we recruited 28 pediatric uveitis patients; 3 were excluded due to unclear images, so the study included 25 cases and 21 healthy children. Patients were recruited from the Department of Ophthalmology outpatient settings and referred to the Department of Pediatric Rheumatology and the NFC unit in the Department of Rheumatology, Rehabilitation, and Physical Medicine for clinical and NFC evaluation at a tertiary hospital.

Ethical approval was obtained from the local Faculty of Medicine Ethical Committee (IRB No.: 04–2023-300337). Informed written consent was obtained from the parent/legal guardian, and agreement was obtained from the pediatric participants before inclusion in the study. Subjects with evidence of infectious uveitis, nailbed trauma, or any condition that affects microcirculation, such as diabetes mellitus or hypertension, were excluded. Inclusion criteria were age less than 18 years old and definite AU diagnosis. A complete medical history was obtained.

### Ophthalmological evaluation

- Best corrected visual acuity: This was assessed using the Snellen chart through best correction.
- Slit-lamp examination: Examination of the anterior segment included assessing the cornea, anterior chamber angle, and lens (presence of opacities and their grading). Also, the anterior chamber was evaluated carefully with a narrow-slit beam to grade the activity of anterior uveitis according to the number of AC cells. Examination of the vitreous for cells or debris and their grading was done if fundus view is possible. Grading of uveitis was based on SUN criteria (standardization of uveitis nomenclature) [14].
- Intraocular pressure: After placing topical anesthetic, intraocular pressure was measured via rebound tonometry to screen for ocular hypertension or glaucoma.
- Dilated indirect ophthalmoscopy: This included visualization of the macula, optic nerve, retinal vessels, and peripheral retina.
- Ocular ultrasonography: A B-scan ultrasound was performed in cases with hazy media, such as dense cataracts and vitreous haze, to evaluate the posterior segment.

### Rheumatological evaluation

- A comprehensive history was taken, including constitutional, musculoskeletal, and mucocutaneous symptoms.
- Joint examination includes evaluation of signs of arthritis and joint range of motion.

Laboratory investigations, including erythrocyte sedimentation rate (ESR), C-reactive protein (CRP), complete blood count, renal functions, liver enzymes, and antinuclear antibody (ANA), were done.

### Nailfold capillaroscopy technique

**Fig. 1:**
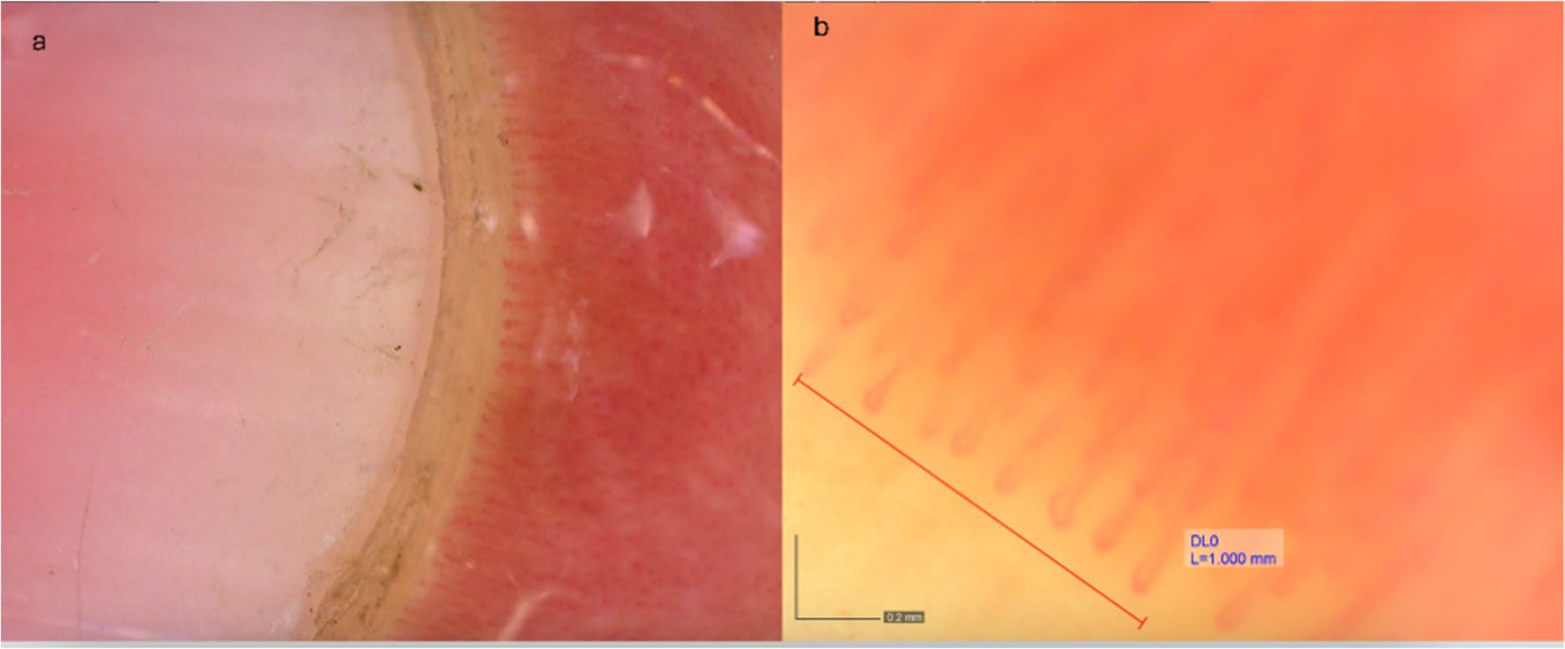
Normal capillary density and distribution at × 50 (**a**) and × 200 (**b**)

**Fig. 2:**
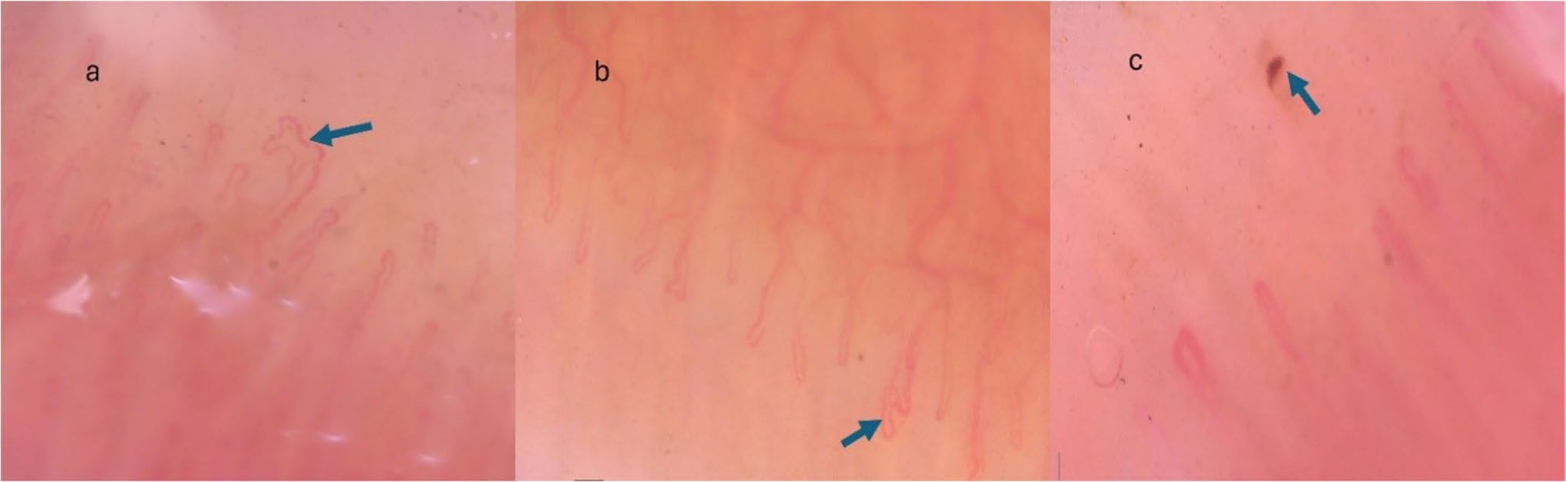
NFC abnormalities at × 200: bizarre-shaped capillary in an 11-year-old male patient with isolated anterior uveitis (**a**), ramified capillary in an 8-year-old male JIA patient with anterior and intermediate uveitis (**b**), and microhemorrhage in a 14-year-old female patient with isolated panuveitis (**c**)

We used a standardized NFC technique from the European Alliance of Associations for Rheumatology (EULAR) study group on Microcirculation in Rheumatic Diseases [15–17]. Dino-Lite digital microscope was used. Each participant was acclimatized for 20 min at room temperature (20–24 °C) before examination. NFC was performed by an experienced rheumatologist. Initially, we applied a drop of immersion oil on nailfolds to enhance resolution. We examined the nailfold of eight fingers (thumbs were excluded). Two images were captured at × 50 magnification to acquire an impression of the nailfold area, as shown in Fig. 1; then, four sequential images were captured at × 200 magnification to assess the capillary density and the detailed capillary morphology and dimensions. DinoCapture 2.0 software version 1.5.48 was applied to analyze images. We evaluate the following NFC parameters at × 200 magnification, as shown in Figs. 1 and 2:

- Capillary density/mm, identified by counting capillary loops in 1 mm in the distal row [18]
- Microhemorrhages, observable as hemosiderin deposits within the distal row [16, 17]
- Avascular areas, labeled as the lack of capillaries in the distal row with a minimum of 200 µm between contiguous capillaries [19]
- Capillary morphology, classified as normal, nonspecific variations, or abnormal (bushy, ramified, or bizarre-shaped) [16, 18]
- Capillary dimensions:
  - The percentage of dilated capillaries (capillaries in a distal row with an apical, efferent, and afferent limb diameter of 20–50 µm)
  - The percentage of giant capillaries (capillaries in a distal row with an apical, efferent, and afferent limb diameter of > 50 µm) [15, 20]

The percentage of each morphology or dimension abnormality was determined by dividing the total number of capillaries of that abnormality by the entire number of capillaries in the distal row per image.

### Statistical analysis

The sample size was calculated via Open Epi V.3.01 software. SPSS (version 26, IBM, USA) was applied to analyze data. Categorical data were expressed as frequencies and percentages, while numerical data were presented as mean ± standard deviation/median and range. Student *T* test was used to compare means among groups when data were normally distributed, while Mann–Whitney *U* test was utilized to compare medians among groups when data were not normally distributed. For comparing categorical data, a chi-square (*χ*^2^) test was performed. Fisher’s exact test was used when the expected frequency was less than 5. The correlation was identified by applying Spearman’s correlation in the case of non-normally distributed data and Pearson’s correlation in the case of normally distributed data. The probability value of less than 0.05 was used as a cut-off point for considering the significance of the statistical test. All tests were two-tailed.

## Results

This study included 25 AU patients and 21 age and gender-matched healthy children with mean ages of 11.24 ± 3.03 and 9.9 ± 4.17 years, respectively, as shown in Table 1. Clinical and laboratory data of uveitis cases are demonstrated in Table 2. Most included patients had isolated uveitis (64%). The most prevalent uveitis subtype was anterior uveitis (48%). Negative ANA was detected in 80% of the included cases.

**Table 1:** Socio-demographic data of patients with pediatric uveitis and controls

Variables	Uveitis patients, *n* = 25 (%)	Control group, *n* = 21 (%)	*p*-value
**Age (years),** mean ± SD (range)	11.24 ± 3.03 (7–16)	9.9 ± 4.17 (4–16)	0.22*
**Gender**			
▪ Male	18 (72%)	11 (52.38%)	0.17**
▪ Female	7 (28%)	10 (47.62%)	
**Anthropometric measures**			
▪ Body mass index (Kg/m^2^)	20.95 ± 4.57	19.84 ± 4.09	0.4*

Data expressed as mean ± SD (range) or frequency (%)

^*^Independent sample *T* test; **chi-square test. The *p*-value was significant if < 0.05

**Table 2:** Clinical and laboratory characteristics of pediatric uveitis patients

**Disease duration** (years), median (range)	1 (0.1–10)
**Type of uveitis**	
• Anterior	12 (48%)
• Anterior and intermediate	10 (40%)
• Panuveitis	3 (12%)
**Clinical diagnosis**	
• Isolated	16 (64%)
• JIA	6 (24%)
• Sarcoidosis	1 (4%)
• Behcet’s disease	1 (4%)
• Familial Mediterranean fever	1 (4%)
**Active uveitis**	12 (48%)
**Cases with complications**	14 (56%)
**Laboratory investigations**	
• White blood cell count (× 10^3^/mm^3^)	7.1 ± 2.75
• Red blood cell count (× 10^6^/mm^3^)	4.73 ± 0.41
• Hemoglobin (g/dL)	12.11 ± 1.16
• Platelets (× 10^3^/µL), median (range)	291 (209–510)
• Aspartate transaminase (U/L)	27.1 ± 6.73
• Alanine transaminase (U/L)	20.92 ± 5.84
• Blood urea nitrogen (mmol/L)	2.48 ± 0.72
• Serum creatinine (µmol/L)	61 ± 13
• First hour ESR (mm/h), median (range)	23 (7–75)
• CRP (mg/dL), median (range)	5 (0–30)
**ANA**	
• Positive	5 (20%)
• Negative	20 (80%)

Data expressed as mean ± SD/median (range) or frequency (%)

*JIA* juvenile idiopathic arthritis, *ESR* erythrocyte sedimentation rate, *CRP* C-reactive protein, *ANA* antinuclear antibody

Therapeutic history included methotrexate, leflunomide, azathioprine, cyclosporine, and prednisolone. In addition, biologic therapy (adalimumab) was taken by seven patients (28%).

Interpreting nailfold capillaroscopic parameters (Table 3), a significant difference was found between cases and controls regarding mean capillary density (6.97 ± 0.85 versus 7.94 ± 0.83, *p* = 0.0003) and the number of subjects having a capillary density less than 7 (13 (52%) versus 2 (9.52%), respectively, *p* = 0.002). The most observed abnormal morphology was the ramified subtype in nine cases, 36% versus seven controls, 33.33% (*p* = 0.755). Capillaries with abnormal dimensions were observed in six patients (24%) versus one healthy control (4.76%) (*p* = 0.11). Other capillaroscopic abnormalities did not show any significant difference between the studied groups. We detected microhemorrhages in seven subjects (six cases and one healthy control) with no significant difference (*p* = 0.11). Only three cases had avascular areas.

**Table 3:** Nailfold capillaroscopic findings in uveitis and control groups

Nailfold capillaroscopic parameters	Uveitis, *n* = 25	Pediatric controls, *n* = 21	*p*-value
Mean capillary density/mm	6.97 ± 0.85	7.94 ± 0.83	0.0003*
Capillary density < 7/mm, *n* (%)	13 (52%)	2 (9.52%)	0.002**
Microhemorrhages, *n* (%)	6 (24%)	1(4.76%)	0.11****
Avascular areas, *n* (%)	3 (12%)	0 (0)	0.239****
Subjects having capillaries with abnormal morphology, *n* (%)	10 (40%)	7 (33.3%)	0.641 **
• Subjects having bushy capillaries, *n* (%)	3 (12%)	0 (0%)	0.24 ****
• Subjects having bizarre-shaped capillaries, *n* (%)	2 (8%)	1 (4.76%)	0.7**
• Subjects having ramified capillaries, *n* (%)	9 (36%)	7 (33.33%)	0.85**
• Bushy capillaries %^a^, median (range)	0 (0.1.25)	0 (0)	0.105***
• Bizarre shape capillaries %^a^, median (range)	0 (0–1.25)	0 (0–4.13)	0.599***
• Ramified capillaries %^a^, median (range)	0 (0–8.31)	0 (0–8.25)	0.755***
Subjects having capillaries with abnormal dimensions, *n* (%)	6 (24%)	1 (4.76)	0.11****
• Subjects having dilated capillaries, *n* (%)	4 (16%)	1 (4.76%)	0.357**
• Subjects having giant capillaries, *n* (%)	4 (16%)	0 (0%)	0.11****
• Dilated capillaries %^a^, median (range)	0 (0–4.38)	0 (0–0.63)	0.229***
• Giant capillaries %^a^, median (range)	0 (0–4.38)	0 (0)	0.058***

Data expressed as mean ± SD/median (range) or frequency (%)

^*^Independent sample *T* test; **chi-square test; ***Mann–Whitney *U* test; ****Fisher’s exact test. The *p*-value was significant if < 0.05

^a^The percentage of each morphology and dimension abnormality was calculated by dividing the sum of the capillaries with that morphology or dimension abnormality by the total number of capillaries evaluated per image

We could not detect significant differences between isolated uveitis cases and those with uveitis associated with systemic disease regarding all capillaroscopic parameters. Also, the presence of complications or active uveitis did not differ significantly in capillaroscopic findings. Capillaroscopic findings did not show a significant difference between males and females.

Our results did not demonstrate a significant difference between patients receiving biologics and those without biological therapy.

Mean capillary density did not show a significant correlation with age, disease duration, or acute phase reactants, as demonstrated in Table 4.

**Table 4:** Correlation of capillary density with different variables

Variable	*R*	*p*-value
Age	− 0.017	0.937**
Disease duration	− 0.075	0.722*
ESR	− 0.04	0.85*
CRP	0.054	0.798*

*R* correlation coefficient, *ESR* erythrocyte sedimentation rate, *CRP* C-reactive protein

^*^Spearman’s correlation; **Pearson’s correlation

## Discussion

In this study, we hypothesize systemic microcirculation involvement in pediatric AU. Our results revealed a significant difference between pediatric AU cases and healthy children regarding mean capillary density and the number of subjects with low capillary density (< 7 capillaries/mm). We detected 13 cases (52%) and 2 controls (9.52%) having low capillary density. A previous study concluded that capillary density is similar in healthy children and adult subjects (≥ 7 capillaries/mm) [17]. In agreement with our results, a previous case–control study in adults with uveitis found significantly lower capillary density in the patients’ group (*p* < 0.001) [19].

In accordance with our results, Kouwenberg and colleagues could not detect a significant difference between pediatric uveitis cases and controls regarding the presence of microhemorrhages (*p* = 0.97) and percentage of bizarre and bushy capillaries (*p* = 0.17 and 0.23, respectively). The same study could not detect a significant difference regarding capillary density between cases and controls (*p* = 0.05); however, they observed significantly lower mean capillary density in the anterior uveitis group (7.0 ± 0.7) compared to the control group (7.5 ± 0.7, *p* = 0.008) [23]. To our knowledge, this is the only study that investigated NFC findings in pediatric AU.

A case–control study included adults with Bechet’s disease showed a significantly higher frequency of ramified capillaries in cases with posterior segment involvement (*p* = 0.015) compared to those without posterior segment involvement [24], which may indicate that autoimmune ocular disease could be associated with microcirculation abnormalities.

Our results indicate systemic microcirculation involvement in isolated uveitis with no sex difference. So, isolated uveitis is not only an intraocular condition but can also include systemic involvement. A previous study included subjects with JIA uveitis, JIA without eye involvement, idiopathic anterior uveitis, and healthy children (*n* = 18, 11, 12, and 11, respectively) and concluded that frequency of interleukin (IL)-13-expressing cells and IL-13 and IL-10 expression levels were elevated in all patient subgroups, while percentages of IL-5-expressing T cells were diminished. In addition, proinflammatory Th17 cells and T cells expressing CTLA-4 were elevated in all patient subgroups, while γ/δ T cells were reduced. Similar results were observed in JIA and idiopathic anterior uveitis groups [25].

## Conclusions

The current findings propose that AU is not only an intraocular inflammation but may also be accompanied by systemic microcirculation involvement, even in cases with isolated uveitis. AU and alterations of the microcirculation may share a common etiopathogenesis. More studies are required to clarify the connection between NFC abnormalities and different uveitis subtypes and factors contributing to these abnormalities. Also, observation of the progression of these abnormalities and response to treatment should be evaluated.

## Limitations

- Limited sample size
- There were few cases with posterior segment involvement, so we could not compare NFC findings between cases with anterior and posterior segment involvement

## Data Availability

Data is available from the corresponding author upon reasonable request.

## Abbreviations

ANA: Antinuclear Antibody
AU: Autoimmune uveitis
CRP: C-reactive protein
E.S.R: Erythrocyte sedimentation rate
EULAR: European Alliance of Associations for Rheumatology
IL: Interleukin
JIA: Juvenile idiopathic arthritis
NFC: Nailfold capillaroscopy
